# The thermal conductivity of the Earth's core and implications for its thermal and compositional evolution

**DOI:** 10.1093/nsr/nwaa303

**Published:** 2020-12-26

**Authors:** Kenji Ohta, Kei Hirose

**Affiliations:** Department of Earth and Planetary Sciences, Tokyo Institute of Technology, Japan; Earth-Life Science Institute, Tokyo Institute of Technology, Japan; Department of Earth and Planetary Science, The University of Tokyo, Japan

## Abstract

Determining the thermal conductivity of iron alloys at high pressures and temperatures are essential for understanding the thermal history and dynamics of the Earth's metallic cores. The authors summarize relevant high-pressure experiments using a diamond-anvil cell and discuss implications of high core conductivity for its thermal and compositional evolution.

The thermal conductivity of iron alloys is a key to understanding the mechanism of convection in the Earth's liquid core and its thermal history. The Earth's magnetic field is formed by a dynamo action that requires convection in the liquid core. Present-day outer core convection can be driven by the buoyancy of light-element-enriched liquid that is released upon inner core solidification in addition to thermal buoyancy associated with secular cooling. In contrast, before the birth of the inner core, the core heat loss must be more than the heat conducted down the isentropic gradient in order to drive convection by thermal buoyancy alone, which can be a tight constraint upon the core thermal evolution.

Recent mineral physics studies throw the traditional value of the Earth's core thermal conductivity into doubt (Fig. 1). Conventionally the thermal conductivity of the outer core had been considered to be ∼30 W m^−1^ K^−1^, an estimate based on shock experiments and simple physical models including the Wiedemann-Franz law: *κ*_el_ = *LTρ*^−1^, where *κ*_el_, *L, T* and *ρ* are electronic thermal conductivity, Lorenz number, temperature and electrical resistivity, respectively [1]. Such relatively low core conductivity indicates that liquid core convection could have been driven thermally even with relatively slow cooling rate. However, in 2012–2013, our conventional view was challenged by both computational and experimental studies showing much higher core conductivity [2–4].

**Figure 1.**
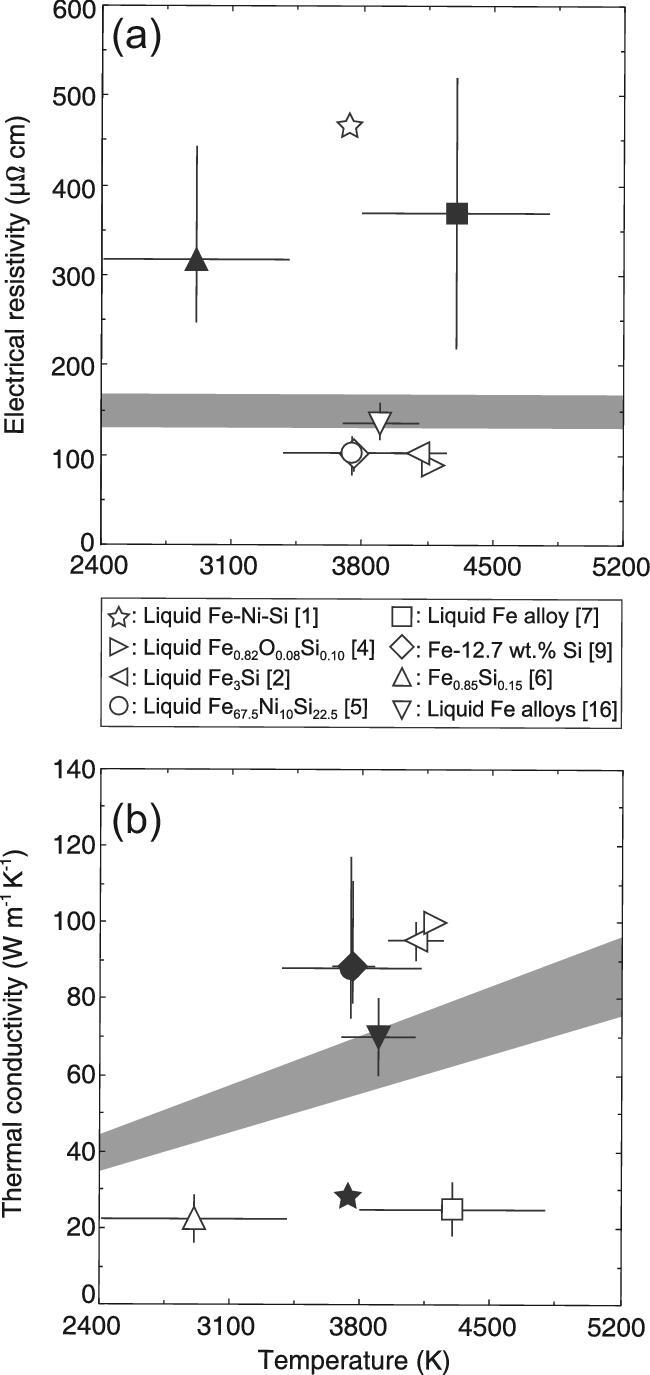
(a) Electrical resistivity and (b) thermal conductivity values at the top of the Earth's core in the literature [1,2,4–7,9,16]. Filled symbols were calculated on the basis of the Wiedemann-Franz law with ideal Lorenz number (*L*_0_ = 2.44 × 10^−8^ W Ω K^−2^). Gray bands indicate (a) the range of saturation resistivity [9] and (b) thermal conductivity computed from the saturation resistivity and the Wiedemann-Franz law.

Since then, experimental determinations of the thermal conductivity of iron and alloys have been controversial (Fig. 1). Ohta *et al.* [5] measured the electrical resistivity of iron under core conditions in a laser-heated diamond-anvil cell (DAC). The results demonstrate relatively high thermal conductivity of ∼90 W m^−1^ K^−1^ for liquid Fe-Ni-Si alloy based on their measured resistivity for pure iron, Matthissen's rule and Wiedemann-Franz law, which is compatible with *ab initio* simulations [2,4]. On the other hand, flash laser-heating and fast thermal radiation detection experiments demonstrated the low core conductivity of 20–35 W m^−1^ K^−1^ based on finite element method simulations [6,7], in accordance with the traditional estimate [1]. Since transport properties that describe non-equilibrium phenomena are difficult to measure, the fact that determinations of the iron conductivity under core conditions have become viable these days is a remarkable success in mineral physics. Nevertheless, the discrepancy in core conductivity makes a big difference in the expected age of the inner core, mechanism of liquid core convection and thermal history [3].

Despite a number of subsequent studies based on a variety of different techniques, we still see a dichotomy of proposed core conductivity values (Fig. 1). The ‘saturation’ resistivity, which is derived from the fact that the mean free path of electron–phonon interaction cannot be longer than the interatomic distance, gives the lower bound for conductivity. Such saturation resistivity lies between two clusters of reported high and low resistivity values. While the resistivity saturation is important in highly resistive transition metals and their alloys [3,8] (Fig. 2), the conventional estimate [1] did not include the effect of saturation in their models, which resulted in much higher resistivity than the saturation value and hence low core conductivity. The core electrical resistivity measured by recent DAC experiments [3,5,9] shows resistivity saturation (Fig. 2), demonstrating the high core conductivity as far as the Wiedemann-Franz law holds with ideal Lorenz number (Fig. 1). Additionally, since temperature has a large effect on resistivity, temperature gradient in a laser-heated sample is an issue. An internally-resistance-heated DAC provides homogenous and stable sample heating and is thus a promising technique for conductivity measurements at high pressure and temperature (*P–T*) [9]. The validity of the Wiedemann-Franz law under extreme conditions has also been an issue. Simultaneous measurements of the electrical resistivity and the thermal conductivity of iron alloy under core high *P–T* conditions will provide decisive evidence for it.

**Figure 2.**
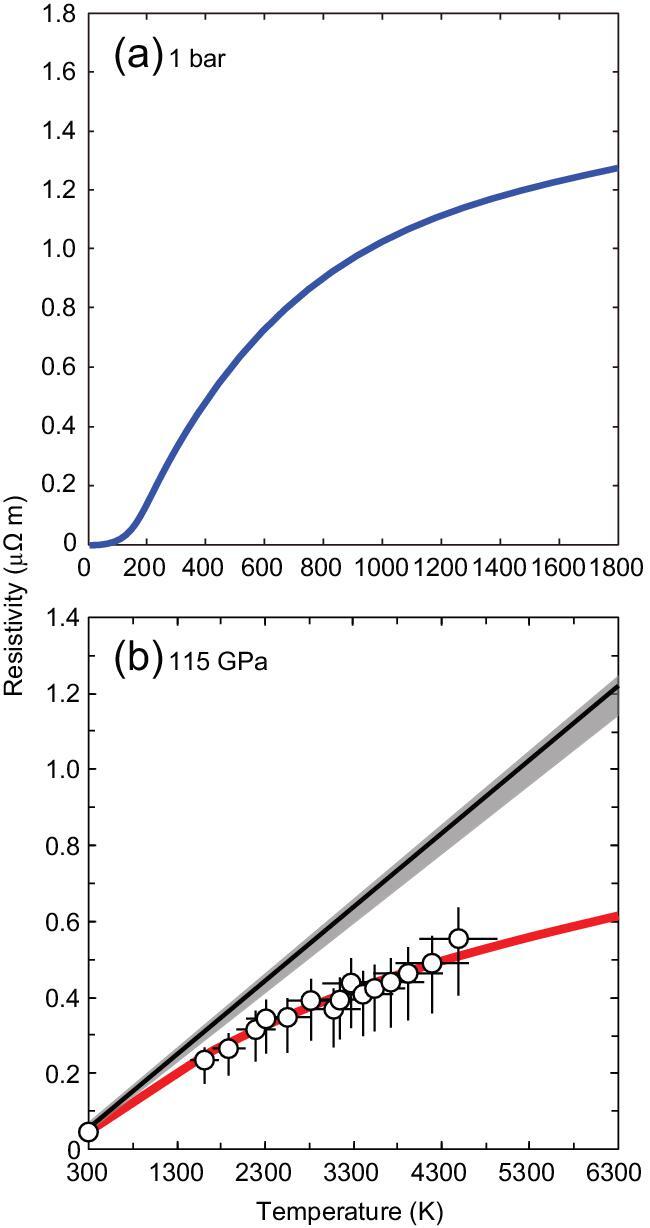
Temperature response of the electrical resistivity of (a) fcc iron estimated at 1 bar [8] (blue curve) and (b) hcp iron at 115 GPa [5]. Red curve and black line with gray uncertainty band indicate the predicted resistivity based on the Bloch-Grüneisen model with and without the resistivity saturation, respectively.

As introduced above, the most recent high *P–T* measurements for Fe containing 2, 4, 6.5 wt.% Si using an internally-resistance-heated DAC have demonstrated that the thermal conductivity of Fe-12.7 wt.% (22.5 at.%) Si is ∼88 W m^−1^ K^−1^ at core-mantle boundary (CMB) conditions when the effects of resistivity saturation, melting and crystallographic anisotropy at measurements are taken into account [9] (Fig. 1). Thermal conductivity of Fe-10 at.% Ni-22.5 at.% Si alloy, a possible outer core composition, could be ∼79 W m^−1^ K^−1^ considering the impurity effect of Ni [10]. Si exhibits the largest ‘impurity resistivity’, indicating that the 79 W m^−1^ K^−1^ is the lower bound for the thermal conductivity of the Earth's liquid core. The core thermal evolution models by Labrosse [11] demonstrated that if liquid core convection has been driven by thermal buoyancy with the core thermal conductivity of 79 W m^−1^ K^−1^ at the CMB and no radiogenic heating in the core, the CMB temperature is calculated to be ∼5500 K at 3.2 Ga and ∼4800 K at 2.0 Ga. Such high CMB temperature suggests that the whole mantle was fully molten until 2.0–3.2 Ga. It is not consistent with geological records, calling for a different mechanism of core convection.

Chemical buoyancy may be an alternate means of driving convection in the core from the early history of the Earth. It has been proposed that the compositional buoyancy in the core could arise from the exsolution of MgO, SiO_2_ or both [12–14]. Recent core formation models based on the core-mantle distributions of siderophile elements suggest that core metals segregated from silicate at high temperatures, typically at 3000–4000 K and possibly higher [13,15], which enhances the incorporation of lithophile elements including Si and O, and possibly Mg into metals. It is suggested that the (Si, O)-rich liquid core may have become saturated with SiO_2_ upon secular cooling [14]. Indeed, the original core compositions proposed in recent core formation models include Si and O beyond the saturation limit at CMB conditions [15], i.e. 136 GPa and 4000 K, leading to SiO_2_ crystallization [13]. The rate of SiO_2_ crystallization required to sustain geodynamo is as low as 1 wt.% per 10^9^ years, which corresponds to a cooling rate of 100–200 K Gyr^−1^ [14]. The most recent model of the core compositional evolution by Helffrich *et al.* [13] showed that MgO saturation follows SiO_2_ saturation only when >1.7 wt.% Mg in the core. If this is the case, in addition to solid SiO_2_, (Mg, Fe)-silicate melts exsolve from the core and transfer core-hosted elements such as Mo, W and Pt to the mantle. The core-derived silicate melts may have evolved toward FeO-rich compositions and now represent the ultra-low velocity zones above the CMB.

## References

[1] Stacey FD , Loper DE. Phys Earth Planet Inter 2007; 161: 13–8. 10.1016/j.pepi.2006.12.001

[2] de Koker N , Steinle-Neumann G, Vlcek V. Proc Natl Acad Sci USA 2012; 109: 4070–3. 10.1073/pnas.1111841109 22375035 PMC3306690

[3] Gomi H , Ohta K, Hirose K et al. Phys Earth Planet Inter 2013; 224: 88–103. 10.1016/j.pepi.2013.07.010

[4] Pozzo M , Davies C, Gubbins D et al. Nature 2012; 485: 355–8. 10.1038/nature11031 22495307

[5] Ohta K , Kuwayama Y, Hirose K et al. Nature 2016; 534: 95–8. 10.1038/nature17957 27251282

[6] Hsieh W-P , Goncharov AF, Labrosse S et al. Nat Commun 2020; 11: 3332. 10.1038/s41467-020-17106-7 32620830 PMC7335046

[7] Konopkova Z , McWilliams RS, Gomez-Perez N et al. Nature 2016; 534: 99–101. 10.1038/nature18009 27251283

[8] Bohnenkamp U , Sandström R, Grimvall G. J Appl Phys 2002; 92: 4402–7. 10.1063/1.1502182

[9] Inoue H , Suehiro S, Ohta K et al. Earth Planet Sci Lett 2020; 543: 116357. 10.1016/j.epsl.2020.116357

[10] Gomi H , Hirose K, Akai H et al. Earth Planet Sci Lett 2016; 451: 51–61. 10.1016/j.epsl.2016.07.011

[11] Labrosse S . Phys Earth Planet Inter 2015; 247: 36–55. 10.1016/j.pepi.2015.02.002

[12] O’Rourke JG , Stevenson DJ. Nature 2016; 529: 387–9. 10.1038/nature16495 26791727

[13] Helffrich G , Hirose K, Nomura R. Geophys Res Lett 2020; 47: e2020GL089218. 10.1029/2020GL089218

[14] Hirose K , Morard G, Sinmyo R et al. Nature 2017; 543: 99–102. 10.1038/nature21367 28225759

[15] Fischer RA , Nakajima Y, Campbell AJ et al. Geochim Cosmochim Acta 2015; 167: 177–94. 10.1016/j.gca.2015.06.026

[16] Zhang Y , Hou M, Liu G et al. Phys Rev Lett 2020; 125: 078501. 10.1103/PhysRevLett.125.078501 32857557

